# Recovery 3 and 12 months after hysterectomy: epidemiology and predictors of chronic pain, physical functioning, and global surgical recovery

**DOI:** 10.1097/MD.0000000000003980

**Published:** 2016-07-01

**Authors:** Maurice Theunissen, Madelon L. Peters, Jan Schepers, Jacques W.M. Maas, Fleur Tournois, Hans A. van Suijlekom, Hans-Fritz Gramke, Marco A.E. Marcus

**Affiliations:** a Department of Anesthesiology and Pain Management, Maastricht UMC+; b Department of Clinical Psychological Science, Maastricht University, Maastricht; c Department of Gynecology, Máxima Medical Center, Veldhoven; d Department of Gynaecology, Maastricht UMC+, Maastricht/Orbis Medical Center, Sittard-Geleen; e Department of Anesthesiology and Pain Management, Catharina Hospital, Eindhoven, The Netherlands; f Department of Anesthesiology, ICU, and Perioperative Medicine, Hamad Medical Corporation, Doha, Qatar.

**Keywords:** chronic pain, hysterectomy, physical functioning, postoperative, predictor, recovery

## Abstract

Chronic postsurgical pain (CPSP) is 1 important aspect of surgical recovery. To improve perioperative care and postoperative recovery knowledge on predictors of impaired recovery is essential. The aim of this study is to assess predictors and epidemiological data of CPSP, physical functioning (SF-36PF, 0–100), and global surgical recovery (global surgical recovery index, 0–100%) 3 and 12 months after hysterectomy for benign indication.

A prospective multicenter cohort study was performed. Sociodemographic, somatic, and psychosocial data were assessed in the week before surgery, postoperatively up to day 4, and at 3- and 12-month follow-up. Generalized linear model (CPSP) and linear-mixed model analyses (SF-36PF and global surgical recovery index) were used. Baseline data of 468 patients were collected, 412 (88%) patients provided data for 3-month evaluation and 376 (80%) patients for 12-month evaluation.

After 3 and 12 months, prevalence of CPSP (numeric rating scale ≥ 4, scale 0–10) was 10.2% and 9.0%, respectively, SF-36PF means (SD) were 83.5 (20.0) and 85.9 (20.2), global surgical recovery index 88.1% (15.6) and 93.3% (13.4). Neuropathic pain was reported by 20 (5.0%) patients at 3 months and 14 (3.9%) patients at 12 months. Preoperative pain, surgery-related worries, acute postsurgical pain on day 4, and surgery-related infection were significant predictors of CPSP. Baseline level, participating center, general psychological robustness, indication, acute postsurgical pain, and surgery-related infection were significant predictors of SF-36PF. Predictors of global surgical recovery were baseline expectations, surgery-related worries, American Society of Anesthesiologists classification, type of anesthesia, acute postsurgical pain, and surgery-related infection.

Several predictors were identified for CPSP, physical functioning, and global surgical recovery. Some of the identified factors are modifiable and optimization of patients’ preoperative pain status and psychological condition as well as reduction of acute postsurgical pain and surgery-related infection may lead to improvement of outcome.

## Abstract 2

Supplemental Digital Content is available in the text

## 1 Background

In 2010, 11,697 hysterectomies were performed in women between 20 and 65 years old in the Netherlands.^[1]^ The prevalence of chronic postsurgical pain (CPSP) after hysterectomy is estimated between 5% to 32%^[2]^ or even 50%^[3]^; however, reported numbers are highly influenced by different definitions used for CPSP. But nonetheless, impaired recovery after hysterectomy may have huge social and economic impact.^[4–6]^ Therefore, further knowledge on incidence and risk factors of CPSP after hysterectomy is needed.

CPSP is just 1 aspect of postoperative recovery. Several other aspects may be important for a patient's experience of the success of an operation. Despite many publications on the concept and domains of postoperative recovery, there is no consensus yet about a generally accepted and validated assessment package for the evaluation of postoperative recovery.^[7]^ A study among hysterectomy patients, reflecting on their own recovery, revealed 4 domains: physical symptoms, emotional well-being, activity levels, and decision making.^[8]^ Other researchers described 5 domains of recovery, based on a concept analysis and interview studies among surgery patients and healthcare workers: physical symptoms, physical functions, psychological, social, and activity.^[9–12]^ This article will be based on a framework presented in 2013, proposing core predictor and outcome domains for CPSP in epidemiological studies, according to the IMMPACT recommendations.^[13,14]^ Four outcome domains are suggested: pain, physical functioning, psychological functioning, and global ratings of outcome. Within each domain outcome measures are suggested.

To improve perioperative care and postoperative recovery, knowledge on risk factors of impaired recovery is essential. Currently, many studies have provided insight into predictors and processes involved in chronic pain^[15]^ and other areas of postoperative recovery.^[16,17]^ One of the existing studies on this subject that assessed a subset of the whole range of potential predictors was performed in 2007 at the Maastricht University Medical Center+ (Maastricht UMC+). Predictors of CPSP, functional limitations, global surgical recovery, and quality of life 6 months after various types of surgery were assessed.^[18]^ The most important somatic predictors of suboptimal recovery were a long duration of the operation and high levels of acute postoperative pain. Psychological variables associated with suboptimal long-term outcome were a high level of preoperative fear of surgery and low optimism. However, despite the fact that studies like this can be very indicative, heterogeneity in types of surgery implies the need for further studies to be performed in homogeneous populations. Even in the case of 1 type of surgery under study, heterogeneity in study design may impair the strength of conclusions.^[2]^ To further refine our previous findings in a more homogeneous surgical population, and according to the call for studies on hysterectomy with detailed baseline and follow-up assessments,^[4]^ the present prospective multicenter study was performed.

To obtain a broad view of the patients’ preoperative status, a comprehensive set of demographic, medical, and psychosocial predictor variables was assessed. Of the outcome domains previously mentioned, that is, pain, physical functioning, global surgical recovery, and psychological recovery, this publication will focus on predictors for the first 3 domains. The selection of predictor variables was based on findings from previous studies on CPSP and supplemented with some general background characteristics of patients and routinely obtained medical variables.^[2,15,18–21]^ Important candidate predictors were acute postoperative pain, preoperative pain, and several psychosocial variables. For pre- and postoperative pain, besides the intensity, also location, duration, onset, interference, and the neuropathic character were assessed. The psychosocial variables included both vulnerability and resilience factors. Previous studies have shown that anxiety and pain catastrophizing are among the most important vulnerability factors for CPSP.^[22]^ Other variables that have previously been associated with higher prevalence of CPSP are depression^[23,24]^ and negative outcome expectancies.^[25,26]^ We also included a measure of childhood abuse as this has been related to the prevalence of chronic pain in general^[27]^ and more specifically to gynecological pain.^[28]^ One of the most prominent resilience factors for CPSP appears to be optimism.^[29–31]^ In addition, social support^[32]^ and more general measures of wellbeing^[30,33]^ have been proposed as protective factors. Thus, a comprehensive set of demographic, medical, and psychosocial variables was obtained and assessed for its association with long-term surgical outcome.

The primary aim of this study was to establish somatic and psychosocial risk factors for pain after 3 and 12 months, in Dutch patients undergoing elective hysterectomy. Secondary aims were to establish somatic and psychosocial risk factors for poor physical functioning and global surgical recovery, to assess the prevalence, incidence, and characteristics of chronic pain, and to assess epidemiological data regarding the secondary outcome variables and predictors. Epidemiological data on predictors and outcomes on the 3 domains of recovery will be described for the total population, and those concerning the predictors also separately for patients with and without CPSP. Also, patient-reported complications during follow-up will be described. To provide better insight in the course of recovery, outcome assessment was performed at multiple relevant time points: postoperative day 1 to 4, and at 3 and 12 months following surgery.

## 2 Methods

For this study, approval of the local Medical Ethical Committee's was obtained and all participants gave informed consent. This study was registered at the Dutch Trial Register under number NTR2702 (http://www.trialregister.nl/trialreg/index.asp). This article was written in accordance with the STROBE guidelines.^[34]^

A prospective multicenter cohort study was performed in 4 hospitals in the Netherlands: the Maastricht UMC+ Maastricht, the Catharina Hospital (CzE) Eindhoven, the Máxima Medical Center (MMC) Veldhoven, and from May 2012, the Orbis Medical Center (OMC) Sittard-Geleen. Surgery was performed between September 2010 and January 2014. Patients were admitted to the gynecology or the short stay ward. Data collection took place in the week before surgery, postoperatively up to day 4, and at 3- and 12-month follow-up. Preoperative and follow-up assessments were performed at home by postal questionnaires. Pain was recorded in a pain diary, provided by the study coordinator, until 4 days after the operation. In case of earlier discharge, the diary was continued at home. Pain medication was recorded during the hospital stay only and stopped with discharge. Data on surgery and anesthesia were collected during surgery by the attending anesthesiologist, gynecologist, or study coordinator.

Participants underwent hysterectomy for a benign indication in 1 of the hospitals involved in the study. Patients were selected for inclusion during the preoperative screening visit. Inclusion criteria were informed consent, age between 18 and 65 years, good command of the Dutch language, elective surgery, and total or subtotal hysterectomy with or without oophorectomy. All types and combinations of surgical approach were allowed: vaginal, abdominal, or laparoscopic. Exclusion criteria were history of cancer, illiteracy, and cognitive impairment, as indicated in the medical record or assessed during the informed consent procedure. Patients with a history of malignancy or an oncological indication for surgery were excluded because of potential differences in prognosis as well as perioperative treatment like chemotherapy or radiotherapy that might bias preoperative baseline and predictor data as well as postoperative outcome.

### 2.1 Confounding and data management

Confounding was addressed in several ways. A homogeneous population was selected, that is, females between 18 and 65 years, only hysterectomy as type of surgery, and absence of malignancy. Data were collected prospectively, missing perioperative data were extracted afterward from the medical file, when available. If the pain diary or follow-up questionnaire was not returned, patients received 1 reminder by post. Missing values on multi-item psychosocial questionnaires, physical functioning, and pain interference scale, if <20%, were imputed by the participants’ mean score on that scale. Single-item sociodemographic, surgical, and outcome variables were not imputed. Use was made of validated questionnaires. All questionnaire packages were designed in collaboration with MEMIC Maastricht, center for data and information management. Completed questionnaires were scanned by MEMIC Maastricht and provided directly in SPSS format. Statistically, using multivariate analyses, the collected control and process variables allowed for correction of bias caused by baseline status of outcome and sociodemographic or surgical factors. Finally, all patients who underwent other surgery or reported a malignancy during follow-up were excluded from follow-up analysis.

### 2.2 Baseline and predictor measures

Baseline data consisted of the sociodemographic variables such as age, education level, employment status, marital status, number of children, and gynecological history, including whether women were sexually active or not. Health behavior (smoking and number of active days/week, minimum of 30 min of physical activity), general health status, and comorbidity were assessed using the screening list developed by Statistics Netherlands.^[35]^ American Society of Anesthesiologists (ASA) physical status classification was recorded. Physical functioning was measured with the physical functioning subscale of the RAND health survey short-form 36 (SF-36). This subscale consists of 10 items assessing perceived difficulties in physical activities, range from 0, indicating severely restricted physical activity, to 100 indicating unrestricted physical activity.^[36,37]^

Pre-existing pain was assessed by the Brief Pain Inventory—Short Form (BPI-SF), including location, duration, intensity, intermittence, and pain therapy. The BPI-SF is a 12-item self-administered questionnaire which references pain during the past 24 h. It consists of 2 subscales: pain severity (4 items) and pain interference (7 items). Questionnaire items are scaled from 0 (meaning no pain/no interference) to 10 (meaning worst pain/complete interference).^[38]^ The scale was slightly adapted to assess intensity of both hysterectomy-related (i.e., pain related to the planned hysterectomy and gynecological pain) and nonhysterectomy-related pain over the last week instead of the last 24 h. We added 1 item to assess “any pain” at the moment of completion of the questionnaire, using the numeric rating scale (NRS) 0 to 10. In order to further characterize the pain, the Douleur neuropathique 4 (DN4) was administered. The DN4 is a 10-item screening instrument to assess the neuropathic character of the pain. Because examination at home by study team members was not possible, only the 7 self-reported items on neuropathic pain (NPP) could be applied, the DN4-interview. When using this 7-item variant of the instrument, a score ≥ 3 indicates presence of NPP, with a sensitivity of 78% and specificity of 81%.^[39]^ In case of missing data on the DN4-interview scale, NPP was considered present if at least 3 positive scores were completed on the DN4-interview scale, or absent in the case of at least 5 negative scores.

Collected surgery-related data were type of incision (median lower abdominal, Pfannenstiel, vaginal, laparoscopic hysterectomy [LH], or laparoscopic assisted vaginal hysterectomy [LAVH]), total or subtotal hysterectomy with or without oophorectomy, indication for the operation, type of anesthesia (general [GA], spinal, GA combined with epidural or spinal), postoperative analgesics, duration of operation, blood loss, complications, conversion of incision type, hospital where the operation took place, and experience of the attending gynecologist expressed as years of training including fellowship. Acute postsurgical pain was assessed until day 4. Every evening patients reported pain at rest and at movement, highest and average pain over the last 24 h (NRS 0–10), use of pain medication yes/no, and at day 4 also NPP.

The psychological predictors included were outcome expectancy, surgical fear, pain catastrophizing, optimism, social support, depression, well-being, and childhood abuse. Outcome expectancies were measured on the basis of 3 items: expected pain intensity at postoperative day 4,^[25]^ expected level of surgical recovery 3 months after hysterectomy assessed with the global surgical recovery index,^[40]^ and expected time until full return to work/normal activities.^[25]^ The first 2 items were chosen so as to match the corresponding outcome domains assessed in this study. As previously noted, expectancy measures whose domain of behavior matches that of the outcome will have the highest predictive value.^[41,42]^ In addition, expectations about the effect of hysterectomy on feelings of femininity and whether hysterectomy would mean a relief or a loss^[43,44]^ were assessed on the basis of 2 tailored items. Surgical fear was measured by using the 8-item Surgical Fear Questionnaire (SFQ), whose validation was recently described.^[45]^ Pain catastrophizing was measured with the Dutch version of the Pain Catastrophizing Scale (PCS). The PCS consists of 13 items assessing an exaggerated negative interpretation of the meaning of pain. It has good reliability and validity.^[46]^

Optimism/pessimism was assessed by the revised Life Orientation Test (LOT-R). The LOT-R has 10 items, 3 of which measure a positive outlook on the future and 3 items measure a negative outlook. After reversal of the negative items, a single-optimism score can be obtained.^[47]^ Social support was measured with the Dutch version of the Medical Outcomes Study—social support survey (MOS-SSS).^[48]^ This is a 19-item self-report questionnaire with 4 support subscales: emotional/informational, tangible, positive interaction, and affectionate. We present the overall support index and the number of close friends/relatives available for support. Depression was measured with the Center for Epidemiological Studies—Depression (CES-D) questionnaire.^[49]^ This self-report instrument is developed for the assessment of depressive symptoms in the general population. Psychological well-being was assessed with the 12-Item Well-Being Questionnaire (W-BQ12).^[50]^ The W-BQ12 provides a brief measure of positive well-being, energy and negative well-being. The W-BQ12 avoids the use of somatic items, and is therefore particularly suitable for use in patient populations. The negative well-being subscale of W-BQ12 was omitted to avoid overlap with the CES-D. Childhood physical or sexual abuse was assessed using a validated 2-item screening questionnaire.^[51]^

### 2.3 Outcome measures

Predictors were assessed for 3 outcomes of postoperative recovery at 3- and 12-month follow-up. For the primary outcome, CPSP, the BPI-SF was used. Patients first had to answer a question about whether they had pain related to the surgical procedure or not. If so, the BPI-SF had to be completed. In the case of absence of hysterectomy-related pain, they could continue with the next section of the questionnaire and the pain scores were replaced by 0. Predictor analysis was performed with 1 of the 4 pain severity items, the highest hysterectomy-related pain score during the last week, NRS 0 to 10. In accordance with clinical practice and previous CPSP studies, the NRS score was dichotomized into the no-CPSP group, NRS 0 to 3 indicating no or slight pain, and the CPSP group, NRS 4 to 10 indicating moderate to severe pain.^[52,53]^ The second outcome measure used for predictor analysis was SF-36 physical functioning, range 0 to 100. The third outcome measure in predictor analysis was self-perceived recovery, assessed with the global surgical recovery index. The global surgical recovery index is a generic 1-item scale on which patients score to what extent they feel recovered from surgery. The patients themselves decide which aspects of recovery are most relevant and thus mirrored in their recovery score. The scale ranges from 0%, meaning not recovered at all, to 100%, meaning full recovery. It has been used in previous surgical studies and the correlation with another, more extensive, recovery scale was 0.72.^[18,40]^

In line with the secondary aim, epidemiological data on outcome measures and predictors are presented. For numbers of prevalence and incidence, the scores were dichotomized if applicable. The primary outcome measure pain was dichotomized into the no-CPSP group, NRS from 0 to 3, and the CPSP group, NRS 4 to 10, based on NRS highest hysterectomy-related pain score. In addition to the primary outcome, the neuropathic characters of the pain (DN4-interview), location, onset, and intermittence of the hysterectomy-related pain were assessed. Intensity and location of nonhysterectomy-related pain was also assessed. Patients were asked to report on pain interference, analgesic use, and other forms of pain treatment. Furthermore, health behavior was assessed using the overall physical activity question from the screening list developed by Statistics Netherlands: “How many days per week do you have physical exercise during at least 30 min?”^[35]^ Also, 1 question assessed the number of days after which the patient was capable of performing all activities of daily living again. Finally, the incidence of surgery-related infections, healthcare visits as a result of a complication during follow-up,^[54]^ and the incidence of significant health events, such as an accident, were explored by 3 questions. Data on sexuality, baseline genetics, and psychological recovery were also collected, but to avoid overload of data these results will be published separately at a later stage.

### 2.4 Statistical analyses

We planned to include 500 patients. With an expected loss of 40% (due to either drop-out or exclusion due to reoperations and new malignancies), 300 patients were expected to be available for the follow-up analyses. With a regression analysis with 7 prior covariates (control variables) explaining 20% of variance, alpha set at 0.05 and a power of 90%, this would enable the detection of an R-square increase of 0.027 (R = 0.16). Eventually 517 participants were included, 412 were available for the analyses at 3-month follow-up and 376 at 12-month follow-up.

Data were visually assessed for normality by creating histograms and by Shaphiro–Wilk test. Pain intensity and interference scores are presented as median and interquartile range (25th–75th percentile, IQR) and analyzed using Mann–Whitney *U* test because of non-normal distribution. Other continuous data were tested by Student *t* test and presented as mean and standard deviation (SD). Categorical data are presented as number (%) and analyzed using Chi-squared or Fischer exact test. Baseline and follow-up results are shown for the total study sample. Baseline data and self-reported complications are also shown for the groups CPSP and no CPSP separately, for both 3- and 12-month outcomes, available as Supplementary File (Tables 1B, 2B, 3B, 4B, and 6).

Predictor analysis of the primary outcome pain at 3- and 12-month follow-up was performed on the highest hysterectomy-related pain score during the last week, the no-CPSP group versus the CPSP group. Because of the dichotomized outcome CPSP, generalized linear model analysis was performed using binary logistic regression and furthermore a time variable defining 3- and 12-month follow-up. The results are presented as beta (SD). See Supplementary Table 5B “Final multivariate models: additional legend concerning data reduction and analysis” for detailed information on the statistical analysis. Predictor analyses for the SF-36 physical functioning and global surgical recovery index scores were performed on continuous scores. No clinically accepted cut-off point is available for global surgical recovery index, while the SF-36 physical functioning score can only be interpreted in light of the presurgical SF-36 score. For these outcomes, linear-mixed model analysis with random intercept and a time variable defining 3- and 12-month follow-up was used. Results are presented as estimate (SD), which reflect the change in outcome for each unit of change in the predictor. Goodness-of-fit model is reflected by Akaike Information Criterion (AIC), the lower the better the fit.

To reduce the large number of available predictor variables that could potentially be used in the regression analyses, first bivariate association with the 3 outcomes was assessed. Predictor variables were only entered in the multivariate model if significant at *P* < 0.10 level in bivariate analysis. Furthermore, to maintain a certain uniformity across multivariate analyses of the 3 outcome measures and to further reduce the number of variables, for predictors with a high level of similarity or overlap an a priori choice was made regarding which 1 to retain. This selection was based on congruency between predictor and outcome measures, general applicability or frequent clinical use, or contribution to model fit. See Supplementary Table 5B “Final multivariate models: additional legend concerning data reduction and analysis” for detailed information on the process of data reduction. In the multivariate analyses, type of incision was dichotomized as laparotomy versus vaginal/LH/LAVH. Type of anesthesia was dichotomized as GA versus other, including combination of types. Finally, to reduce the number of psychological predictors a factor analysis with oblimin rotation was performed on the SFQ, PCS, LOT-R, CES-D, and W-BQ12. Principal component analysis revealed 2 factors. The first factor consisted of the SFQ and PCS and was named surgery-related worries. Thus, this factor represents high fear of surgery and high-pain catastrophizing. The second factor consisted of LOT-R, CES-D, and W-BQ12 and was named general psychological robustness. This factor represents a high level of optimism and psychological well-being and a low level of depressive symptoms. Aggregate scores were calculated by summing the weighted scores on the respective questionnaires belonging to the 2 new variables. The MOS-SSS was analyzed separately because, in contrast to the selected 5 psychological variables that reflect intraindividual differences, the MOS-SSS reflects interindividual differences, namely the availability of social support. Also, childhood physical or sexual abuse and outcome expectancies were entered as separate predictors. The outcome expectancy variable corresponding with the particular outcome was used in the multivariate analysis. For the outcome CPSP this was expected pain intensity, and for outcome global surgical recovery this was expected global surgical recovery. For outcome physical functioning, no corresponding baseline expectation was assessed. Expectancies of hysterectomy as a relief or loss and expectancy regarding femininity were used for all outcomes.

The control variables, such as hospital, age, type of incision (dichotomized), type of anesthesia (dichotomized), and the time variable defining 3- and 12-month follow-up, were entered in the multivariate model using a forced entry method. All other predictors were entered using a backward deletion procedure (criterion *P* < 0.10). A final significance level of *P* < 0.05 was used. For each outcome, 2 prediction models are presented. Model 1 reveals the results of multivariate prediction analyses using the control variables supplemented with the significant preoperative predictors. This allows establishing a preoperative risk profile. In model 2, the final model, the pre- and postoperative predictors are added. For all 3 outcome measures, multicollinearity was assessed using the variance inflation factor and tolerance, obtained by entering the predictors used in the multivariate models in a linear regression model. Criteria for absence of multicollinearity are variance inflation factor <10 and average variance inflation factor around 1, tolerance ≥ 0.2. Analyses were performed using IBM SPSS Statistics for Windows, Version 22.0 (Armonk, NY).

## 3 Results

**Figure 1:**
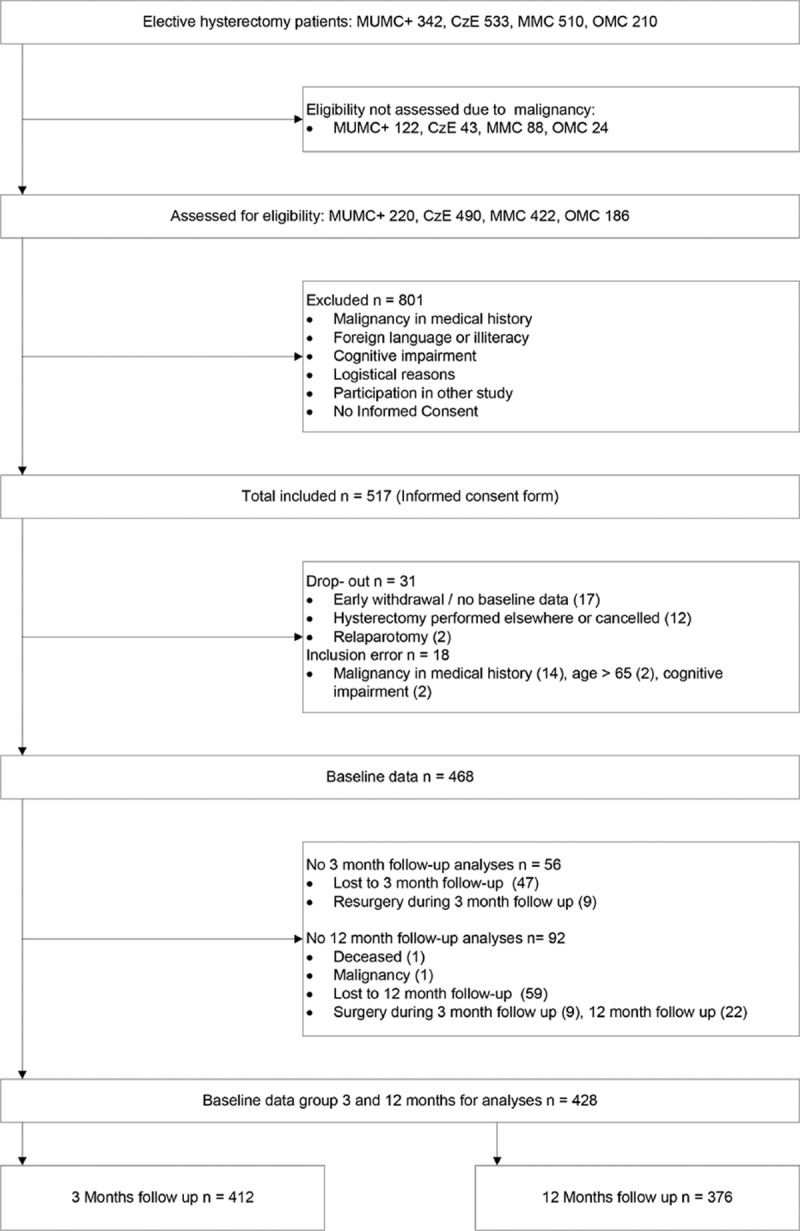
Flow chart patient inclusion.

Patient inclusion is illustrated in the flow chart in Fig. 1. Of the 517 patients providing informed consent, baseline data were obtained from 468 patients. The response rate after 3 months was high with 412 (88%) of 468 operated patients suitable for follow-up evaluation. After 12 months, 376 (80%) patients were suitable for follow-up evaluation. The number of missing data was relatively low: for baseline data range 0% to 12.9%, surgery data 0% to 5.1%, acute pain data 11.2% to 17.3%, and follow-up data 0% to 5.6%. Sociodemographic and baseline health characteristics are presented in Table 1 and 1B (Supplementary File, baseline data split up for the groups CPSP and no CPSP). Psychosocial baseline measures and data concerning surgery and acute postsurgical pain are presented in Tables 2 and 3 (Tables 2B and 3B, Supplementary File). Most commonly applied interventions were vaginal hysterectomy and GA. Preoperative pain characteristics and other physical baseline parameters are presented in Table 4, together with their corresponding outcomes after 3 and 12 months. Similarly to the other baseline characteristics, in Table 4B (Supplementary File), baseline data on pain and physical functioning are presented split up for the groups CPSP and no CPSP at 3 and 12 months, respectively.

**Table 1:**
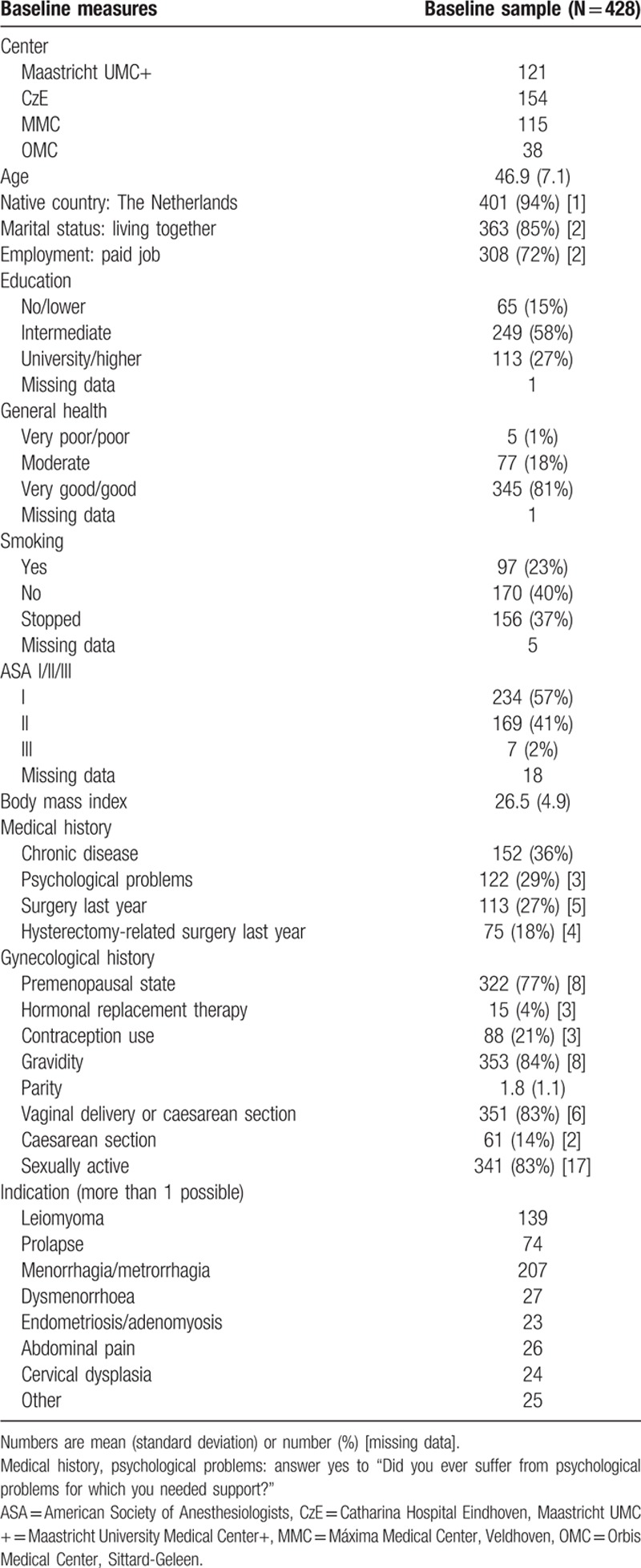
Baseline characteristics.

**Table 2:**
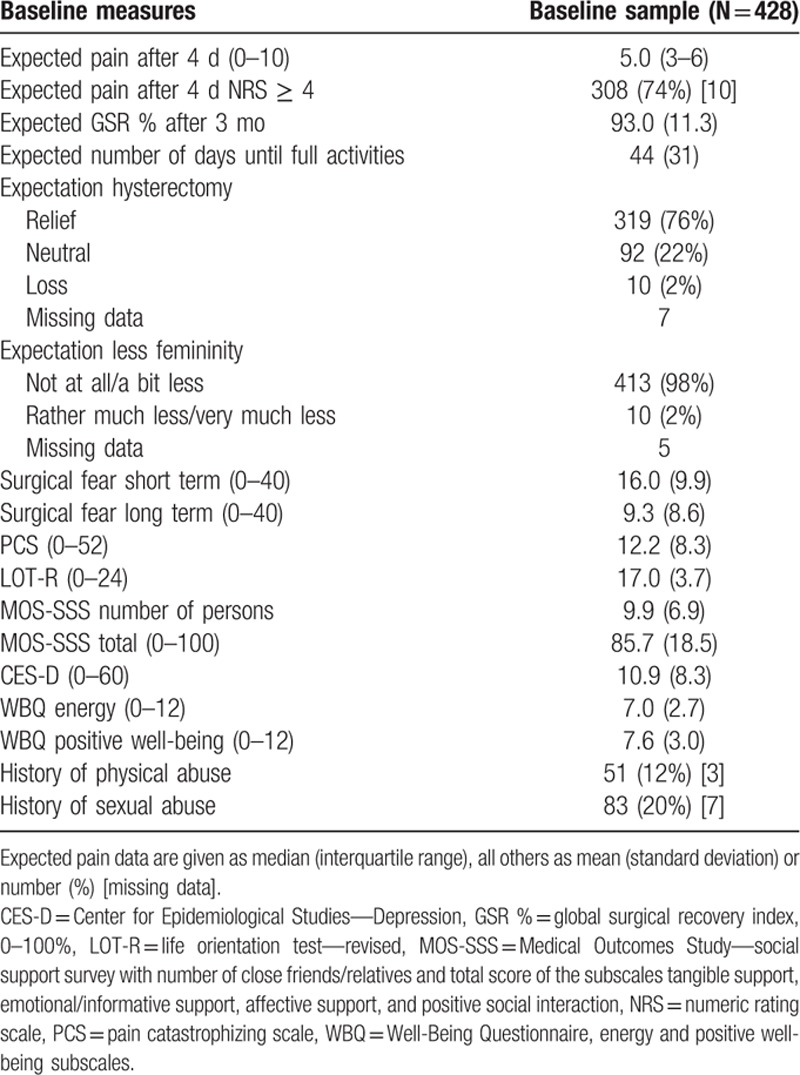
Psychosocial baseline measures.

**Table 3:**
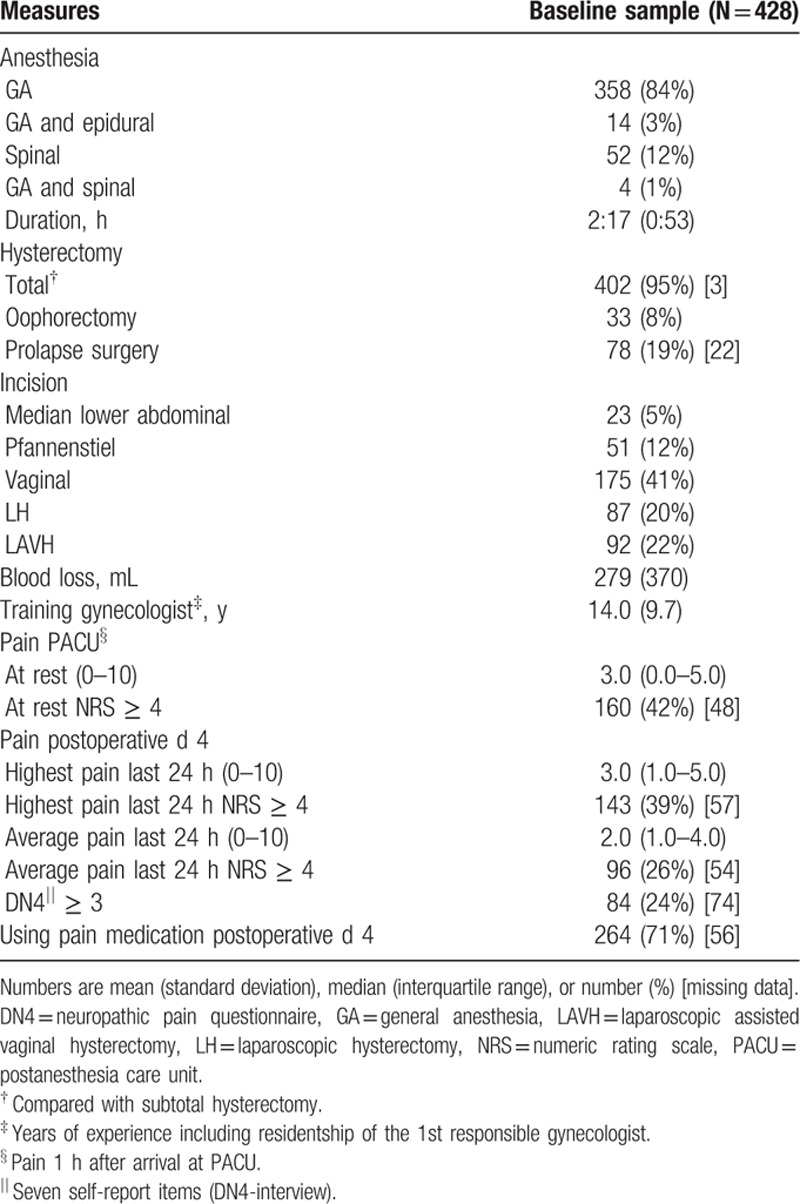
Surgery and acute pain.

**Table 4:**
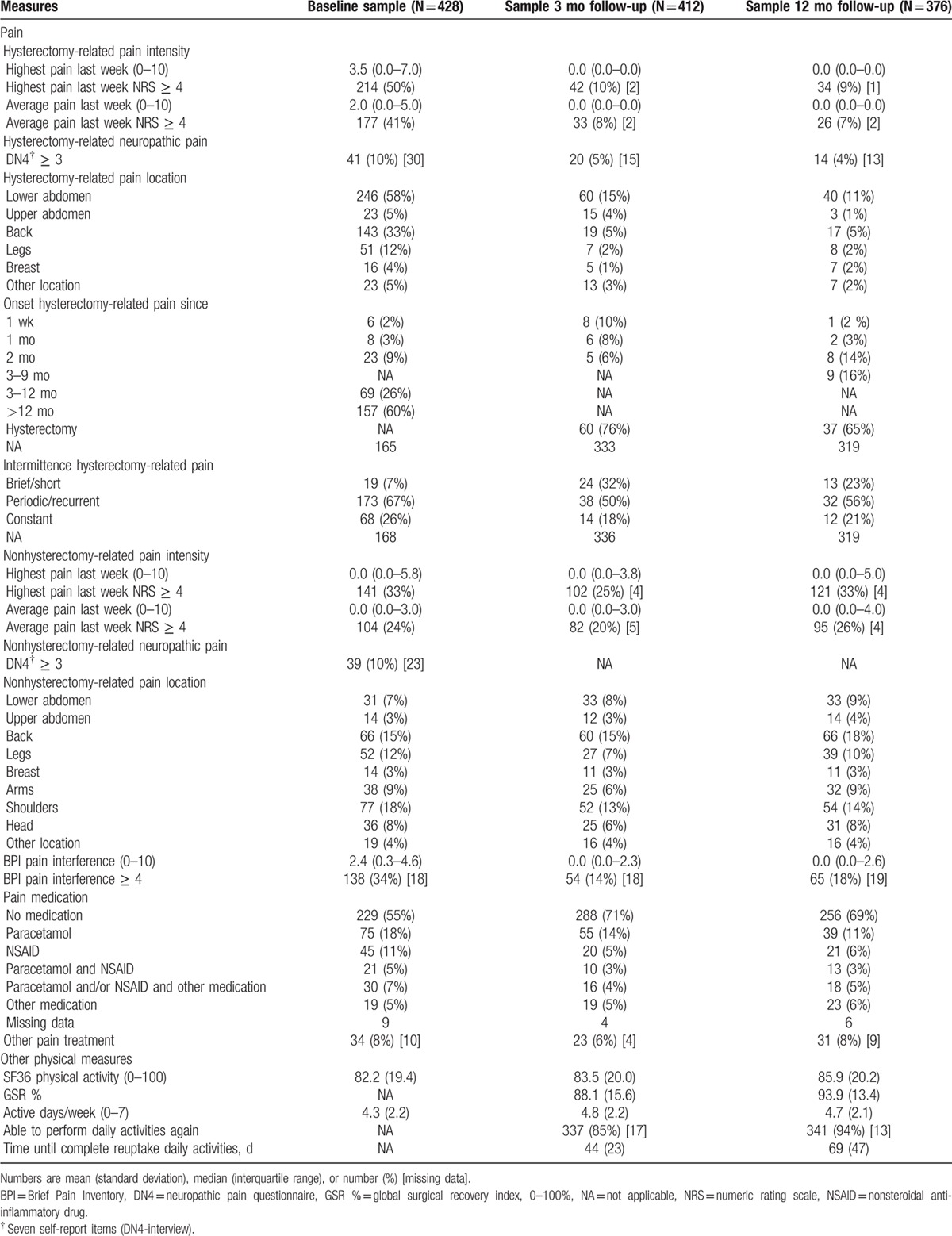
Baseline measures and corresponding recovery scores at 3 and 12 mo after hysterectomy.

### 3.1 Descriptive data of recovery variables

CPSP at 3-month follow-up (hysterectomy-related pain, NRS highest pain last week, cut-off ≥ 4) was indicated by 42 patients (the CPSP group), prevalence 10.2%, 9 of whom had no pain at baseline, incidence 2.2%. In 169 (82.8%) of the patients reporting preoperative hysterectomy-related pain, the pain had disappeared at 3-month follow-up. Median (IQR) pain intensity in the CPSP group was 6 (4.8–7.3) and in the no-CPSP group 0 (0–0) (hysterectomy-related NRS highest pain last week). Nonhysterectomy-related pain at 3 months (NRS highest pain last week, cut-off ≥ 4) was indicated by 102 (25.0%) women. Any pain at 3-month follow-up (hysterectomy- and/or nonhysterectomy-related pain at the moment of completing the questionnaire, NRS cut-off ≥ 4) was indicated by 67 (17.0%) women. An indication of NPP (DN4-interview ≥ 3) was present in 16 patients (45.7%) of the CPSP group versus 4 patients (1.1%) of the no-CPSP group. Occurrence of NPP in relation to type of incision was median lower abdominal 0 (0%), Pfannenstiel 6 (14.0%), vaginal 6 (3.7%), LH 3 (3.7%), and LAVH 5 (5.7%) cases. NPP occurred in 19 patients (5.7%) with GA and in 1 (2.0%) with spinal analgesia. Three months after surgery, 61.9% of the patients in the CPSP group used pain medication as compared with 24.4% of the patients of the no-CPSP group. Pain interference in the CPSP group was 3.9 (2.9–5.6).

At 12 months a minor decrease of CPSP was shown, with 34 women reporting an NRS ≥ 4, prevalence 9.1%. Pain intensity (hysterectomy-related pain, NRS highest pain last week) in the CPSP group was 6 (5–7) and in the no-CPSP group 0 (0–0). Nonhysterectomy-related pain was reported by 121 patients (32.5%), an increase compared with the 3-month results. Any pain at 12-month follow-up (hysterectomy- and/or nonhysterectomy-related pain at the moment of completing the questionnaire) was indicated by 70 (19.7%) women. Hysterectomy-related NPP was reported by 10 women (38.5%) of the CPSP group and 4 women (1.2%) of the no-CPSP group. In the CPSP group, 19 women used analgesia (55.9%) versus 95 (28.4%) of the women in the no-CPSP group. Pain interference in the CPSP group was 3.1 (2.1–6.6).

The mean scores on the SF-36 physical functioning subscale of 83.5 (SD 20.0) at 3-month follow-up appeared to be relatively stable when compared with the preoperative scores of 82.4 (19.3). At 12 months the mean score was 85.9 (20.2).

Self-perceived recovery after 3 months was high with a mean global surgical recovery index score of 88.1 (15.6), but significantly lower than the expected global surgical recovery index of 93.2 (10.8) (*P* < 0.001). However, the expected level was achieved after 12 months of recovery with a mean score of 93.9 (SD 13.4). The time until patients were able to perform their normal daily activities, reported after 3 months, was 44 days. This was in line with their expectations. However, the same question asked at 12 months revealed a mean of 69 days. Data on CPSP, SF-36 physical activity, global surgical recovery, and other indicators of recovery are presented in Table 4. For the outcomes on pain intensity, pain interference, analgesia use, SF-36 physical activity, global surgical recovery, and proportion of women able to perform daily activities again, the scores of patients in the CPSP group are significantly less favorable than for patients in the no-CPSP group, at both 3 and 12 months.

### 3.2 Predictors of recovery after 3 and 12 months

#### 3.2.1 Pain

Of the control variables such as hospital, age, type of anesthesia, and type of incision, only hospital reached significance at 0.10 level in bivariate analysis: CzE beta −0.785, *P* = 0.06, MMC −0.656, *P* = 0.13, OMC −0.073, *P* = 0.98, reference Maastricht UMC+, interaction with time not shown. Besides baseline hysterectomy-related pain, also nonhysterectomy-related preoperative pain, ASA classification, number of pregnancies (gravidity) including interaction with time, expectations about hysterectomy (relief/neutral/loss), expected pain on postoperative day 4, number of close friends/relatives (MOS-SSS), surgery-related worries, general psychological robustness, having undergone prolapse surgery, acute pain at the postanesthesia care unit (PACU), NPP on postoperative day 4, and surgery-related infection during 3- and 12-month follow-up including interaction with time were entered in the multivariate model.

Model 1, based on the control variables including time and supplemented with the preoperative predictors, revealed significant differences in the risk of CPSP between the participating hospitals. Also time (12 months compared with 3 months), baseline hysterectomy-related and nonrelated highest pain, and surgery-related worries were predictors of CPSP. Finally, gravidity decreased the risk of CPSP at 12 months (N = 314, AIC 354.9).

The results of model 2 are presented in Table 5, left column. None of the control variables reached significance anymore. The risk of CPSP was increased by baseline hysterectomy-related and nonrelated highest pain, surgery-related worries, NPP day 4, and infection at 3 months. Although the simple effects of gravidity on CPSP at 3 and 12 months were not significant, beta 0.117, *P* = 0.54 and −0.632, *P* = 0.06, respectively, the time by gravidity interaction was (*P* < 0.05). This is in line with the near-significant effect for the 12-month outcome (N = 253, AIC 287.4).

**Table 5:**
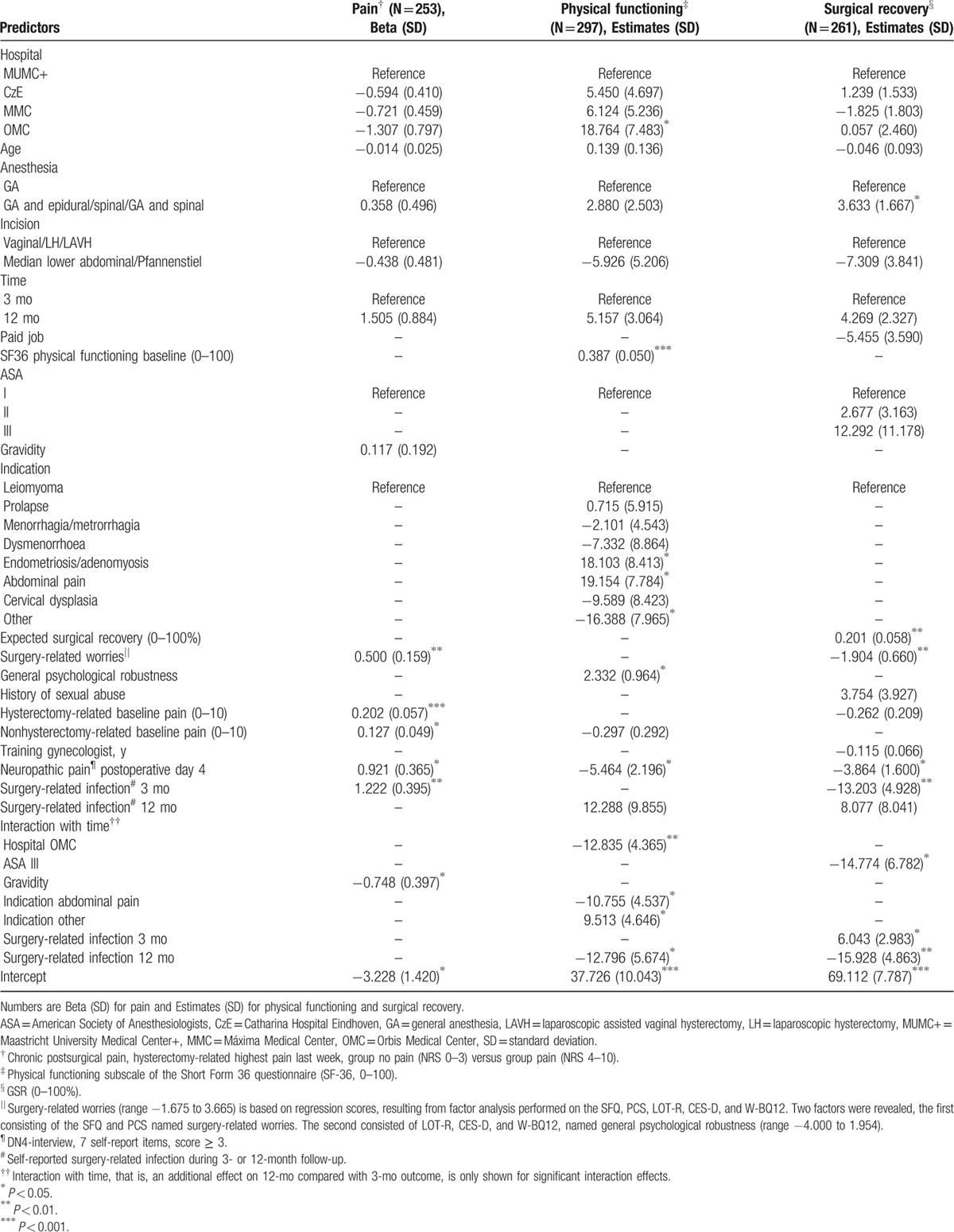
Final multivariate models for predictors of pain, physical functioning, and surgical recovery 3 and 12 mo after hysterectomy.

#### 3.2.2 Physical functioning

For the outcome physical functioning in bivariate analysis, 3 of the control variables were significant at 0.10 level. Hospital, CzE estimate 4.667, *P* = 0.26, MMC 9.530, *P* = 0.03, OMC 7.191, *P* = 0.26, reference Maastricht UMC+. Type of anesthesia, GA with epidural 5.277, *P* = 0.56, spinal 0.407, *P* = 0.94, GA with spinal 58.048, *P* = 0.001, reference GA. Type of incision, median lower abdominal 9.200, *P* = 0.25, Pfannenstiel 13.493, *P* = 0.02, vaginal 9.582, *P* = 0.03, LAVH 0.760, *P* = 0.88, reference LH. Interaction with time not shown. In addition to baseline physical functioning, marital status, employment status (paid job yes/no), ASA classification, expectations about femininity, social support (MOS-SSS total score), the psychological aggregate scores of surgery-related worries and general psychological robustness, baseline hysterectomy-related and nonrelated pain, indication, acute pain at the PACU, NPP day 4, and surgery-related postoperative infection at 12-month follow-up were entered for multivariate analyses.

Model 1, based on the control variables including time and the preoperative predictors, revealed that higher baseline levels of physical functioning and general psychological robustness, and the indication abdominal pain predicted higher postoperative levels of physical functioning. Baseline nonhysterectomy-related pain was a risk factor for lower scores. Concerning the level of physical functioning at 12 months, however, the positive effect of indication abdominal pain was attenuated by a time by indication interaction effect. Furthermore, a time by hospital interaction effect revealed that patients of 1 hospital were at increased risk for lower physical functioning scores at 12 months (N = 411, AIC 6255.8).

Model 2 is shown in Table 5, middle column. It revealed a positive effect on physical functioning outcome for patients of 1 hospital, patients with high baseline physical function and general psychological robustness scores, and patients with indication endometriosis/adenomyosis or abdominal pain. Poorer physical functioning at follow-up was seen with indication “other,” and NPP day 4. Self-reported infection at 12 months predicted lower scores at 12-month follow-up, simple effect estimate −26.101, *P* = 0.007, simple effect at 3-month follow-up 12.288, *P* = 0.21. Interaction effects of time by hospital (*P* = 0.004), time by indication abdominal pain (*P* = 0.02) or indication “other” (*P* = 0.04), and time by self-reported infection at 12 months (*P* = 0.03), indicated an attenuated effect of the corresponding reported simple effects on 12-month follow-up results (N = 297, AIC 4752.9).

#### 3.2.3 Global surgical recovery

Only type of incision was a significant control variable at 0.10 level in bivariate analysis. Reference LH, median lower abdominal estimate −8.380, *P* = 0.20, Pfannenstiel −11.427, *P* = 0.02, vaginal −2.344, *P* = 0.52, LAVH 2.187, *P* = 0.60, interaction with time not shown. Further added to the model were expected global surgical recovery, marital status, employment status, ASA, indication, expectations about hysterectomy, expectations about femininity, history of sexual abuse, the psychological aggregate scores surgery-related worries and general psychological robustness, baseline hysterectomy-related and nonrelated pain, total versus subtotal hysterectomy, experience of the attending gynecologist, NPP day 4, and surgery-related infection during 3- and 12-month follow-up.

Model 1 of the multilevel analyses, based on the control variables including time and the preoperative predictors, revealed that higher global surgical recovery index scores were predicted by higher expected global surgical recovery and history of sexual abuse. Risk factors were type of incision (laparotomy) and increased baseline hysterectomy-related pain and surgery-related worries (N = 383, AIC 5582.8).

Model 2 is presented in Table 5, right column. Baseline global surgical recovery expectations and surgery-related worries remained significant; however, the other predictors of model 1 were exchanged for ASA classification, type of anesthesia, NPP day 4, and surgery-related infection. Patients with combined anesthesia recovered better than patients with GA only. By a significant interaction with time an attenuating effect was shown for patients of ASA class III or reporting an infection at 3 or 12 months. Patients of ASA class III recovered less well at 12 months compared with patients of ASA class I, simple effect with 12-month outcome estimate −32.029, *P* = 0.004, and simple effect with 3-month outcome 12.292, *P* = 0.27. Patients reporting an infection at 3 or 12 months showed poorer recovery at the corresponding time. The simple effect for the 3-month outcome was −13.203, *P* = 0.008, and the simple effect for the 12-month outcome was −39.706, *P* < 0.001 (N = 261, AIC 3961.1).

Multicollinearity did not play any role in the multivariate analyses of the 3 outcomes.

### 3.3 Conversion, complications, and major events during 3- and 12-month follow-up

Conversion of incision type during surgery occurred once in the CPSP group and 12 times in the no-CPSP group (CPSP at 3 months). The incidence of surgical complications such as bleeding, genitourinary tract injury, and gastrointestinal injury was relatively low and in line with recent findings.^[54]^ Blood loss ≥ 1000 mL occurred in 20 patients (4.7%), genitourinary tract injury occurred in 11 patients (2.6%), and gastrointestinal injury was reported in 1 patient (<1%).

A surgery-related infection was reported by 50 (12.4%) patients at 3-month follow-up. Assessed in a different way, 59 (14.3%) patients reported a healthcare visit for reasons of infection or fever, not necessarily surgery-related. At 12 months, these numbers were 17 (4.6%) and 20 (5.3%), respectively. The numbers of patients who contacted their general practitioner, specialist, or the emergency department for different types of complications during follow-up are presented in Table 6 (Supplementary File). Hospitalization during 3-month follow-up was reported by 10 patients, for reasons of: pneumonia, sepsis, wound infection/abscess/hematoma (3); ileus; gastrointestinal complaints, and for flash-backs of incest induced by hysterectomy; urine retention/obstipation, ultimate diagnosis multiple sclerosis; pulmonary embolism; and stroke. At 12 months, reasons for hospitalization were gastroenteritis (2) and stroke.

## 4 Discussion

The primary purpose of the present study was to examine predictors of CPSP, and secondarily of other indicators of recovery, that is, self-reported physical functioning and self-perceived global recovery. CPSP was predicted by the presence of moderate to severe hysterectomy-related and hysterectomy-unrelated pain before the operation and NPP 4 days after the operation. Other predictors were surgery-related worries and surgery-related infection. Interestingly, for the secondary outcome variables, partly different predictors were found. Preoperative pain was not significantly associated with poor physical functioning or global surgical recovery at follow-up. However, abdominal pain as the indication for hysterectomy predicted better physical functioning. Other predictors of physical functioning, besides preoperative physical functioning and indication, were participating center, general psychological robustness, NPP pain at day 4, and surgery-related infection. For global surgical recovery, we identified expected global surgical recovery, surgery-related worries, ASA classification, type of anesthesia, NPP at day 4, and surgery-related infection as predictors.

The prominent role of preoperative pain as predictor of CPSP in the present study is congruent with many other prediction studies across various surgical procedures^[15,55]^ including hysterectomy.^[2,3,56,57]^ We used the highest pain intensity score as predictor instead of the often used mean pain intensity score. Differences were small, but highest pain proved to be the most powerful predictor. Furthermore, there is extensive evidence that high levels of pain in the acute postoperative period are associated a high prevalence of CPSP.^[15,55–57]^ In the present study, we found that especially pain with neuropathic characteristics was associated with CPSP, and additionally with physical functioning and global surgical recovery. The predictive value of surgery-related worries for CPSP is in agreement with previous studies in women undergoing hysterectomy.^[3,56,57]^ Moreover, a systematic review and meta-analysis revealed that preoperative fear and anxiety as well as pain catastrophizing are associated with CPSP across many different interventions.^[22]^ Because of the overlap of these measures, for the current study we created an aggregate measure representing high surgical fear and high pain catastrophizing. Similarly an aggregate score was created for the 3 more global individual difference variables, that is, optimism, depression, and well-being. This aggregate did not prove predictive of CPSP, although 2 of its constituent parts, depression and well-being energy, were significant (*P* < 0.01) in bivariate analyses. In contrast to the findings of several other studies, optimism was not associated with CPSP in our bivariate analyses.^[29–31,33]^ To check whether an aggregate of only depression and well-being would be retained in the multivariate model, we performed a post hoc analysis (data not shown). This truncated aggregate did not reach significance either, suggesting that psychological factors that are more specific for the operation have greater predictive power for CPSP than global psychological states and traits. However, physical functioning was predicted by general psychological robustness. For this outcome, besides depression and well-being energy, the positive well-being subscale was also significant at *P* = 0.01 level. Changes in physical functioning in the course of 1 year may be less directly related to the surgical intervention compared with the other outcomes in this study and additionally determined by the general psychological state of an individual.

Preoperative expectations have also been found predictive of outcome in previous studies.^[25,42,58]^ Here, we found expected global surgical recovery to predict actual global surgical recovery, but expected pain was not predictive of CPSP. Surgery-related infection was a risk factor for physical functioning, global surgical recovery, and also CPSP. Infection may delay healing and affect all aspects of recovery. However, it should be noted that according to the original definition of CPSP, other causes for pain should be excluded.^[59]^ Our data did not allow us to assess whether reported infection was still present at the moment of pain assessment. Therefore we addressed potential confounding effects of infection with regard to outcome CPSP by including infection in the multilevel analyses.

The only other predictor showing significance across all 3 outcomes was NPP. Because NPP at day 4 contributed most to statistical model improvement, it was selected as the index for acute postsurgical pain over NRS pain and analgesia use. The obvious role of NPP suggests that at least part of the problem is persistence of NPP that already starts in the early postoperative period.^[60]^ At 3-month follow-up, the overall rate of NPP was 5% in our study, and 46% of the patients reporting CPSP indicated that their pain had neuropathic characteristics. At day 4, 53% in the CPSP group reported NPP against 19% of patients in the no-CPSP group. Time to recovery of NPP depends on the type of nerve injury.^[61]^ Unfortunately, our data do not allow assessing whether the nerve injury in the no-CPSP group was less severe as compared with patients with CPSP. NPP was reported most after Pfannenstiel incision. Previously it was suggested that indeed Pfannenstiel incisions yield a higher risk of NPP compared with other types of incision.^[62–64]^

The present study provides several clues toward interventions that might reduce the incidence of CPSP after hysterectomy. We presented 2 different predictor models, with the first model including only those variables that can be obtained preoperatively. This may guide the selection of variables to be included in a future risk assessment tool. High-risk patients can be identified before surgery and offered intensified attention by the nursing staff or some form of counseling. A risk assessment tool might also guide treatment decisions regarding the surgical procedure and analgesic regime. The second model, including pre- and postoperative variables as well, gives an even more complete overview of risk factors for long-term outcome and points to the necessity of adequate postoperative pain management and infection prevention. Future studies should also establish the causality of these relations.

The results of this study also show that in general patients recovered well from hysterectomy. The prevalence of moderate to severe hysterectomy-related pain (i.e., NRS ≥ 4) was significantly lower at 3- and 12-month follow-up (10.2% and 9.1%) than it was before the operation (50%). The scores of SF-36 physical functioning, between 82.4 and 85.9, are within the range as measured in female populations in the UK 86.7 (20.2), Australia 85.1 (18.7),^[65,66]^ and a sample of Dutch females 80.4 (24.2).^[36]^ Some of the other study variables also indicated generally good recovery with the number of active days/week increasing significantly from baseline to follow-up with 0.5 days. The number of days until complete reuptake of full activities was within patients’ expectations, at least at 3 months, presumably in line with the prescribed 6 weeks recovery period. Overall, our results are in line with the findings of Linenberger, reporting that most patients described their physical condition 8 weeks after hysterectomy as “better than before.”^[8]^

Despite overall good outcomes, some patients still reported pain after 3 and 12 months. The prevalence of CPSP in our sample was 10.2% to 9.1%, and 3-month incidence 2.2%. These numbers are well within the ranges of 5% to 32% for CPSP and 0% to 15% for acquired/increased pain at follow-up resulting from earlier reports. However, a recent investigation presented a prevalence of even 50% at 4 months after surgery.^[2,3,56,57]^ The high prevalence in the latter study may have resulted from the definition of CPSP, namely any pain at follow-up, whereas the other studies, including the present 1, used more stringent criteria.

This study has several strengths. First, we conducted a large-scale multicenter study with a homogeneous population, that is, females undergoing hysterectomy for benign indications. A comprehensive dataset was assessed allowing for broad analyses of predictors and the course of recovery. For future studies, the results can help to select the most appropriate predictor and outcome measures. Furthermore, based on this extensive baseline and recovery data, preoperative counseling can be further tailored to future patients. Second, compliance was high with 88% and 80% of patients providing follow-up data. Third, we created psychological aggregate scores which might yield more stable outcomes. Many psychological factors are correlated and depending on the specific sample and study, 1 factor might prevail in the multivariate model in one study and another in the next study. But our study still has some limitations. First, to avoid bias patients who underwent resurgery were considered as drop-out. As a result, this study cannot provide insight into causes of resurgery and related complications. Second, NPP was assessed using the 7 self-report items instead of the total DN4. Although validated as DN4-interview, sensitivity and specificity are slightly lower as compared with the 10-item DN4. Third, all psychological assessments were performed by self-assessment. Given the fact that 29% of the patients indicated a history of psychological problems, a clinical diagnosis of current psychopathology would have strengthened our conclusions. Fourth, a large number of baseline data were explored for predictor analyses. As a result, type 1 errors cannot be excluded and inferences with regard to our study population should be made with caution. However, aiming at the development of suitable and comprehensive prediction models for the different outcomes, we notice that despite the risk of type 1 error, narrowing down from a broad scope of potential predictors, many of the established predictors confirmed the results of previous research.

### 4.1 Practical implications

Future interventional studies should aim at further reduction of perioperative pain by using interventions on modifiable psychological and physical factors. This is not only of great clinical relevance, but could provide evidence for the causal status of the proposed predictors. Studies on dedicated acute pain treatment programs would allow evaluating whether patients’ recovery would indeed improve in the case of further postoperative pain reduction. Special attention should be paid to the prevention of NPP. A debate about surgical technique should be initiated among gynecologists, aiming at further reduction of iatrogenic NPP.^[67]^ Additionally, there is growing evidence that besides reduction of acute pain, perioperative treatment with pregabalin or gabapentin might prevent CPSP. This effect is mainly contributed to the efficacy of pregabalin and gabapentin in the treatment of NPP.^[68,69]^ Also other anesthesiological treatment options such as epidural analgesia or patient-controlled analgesia might need reconsideration.^[70]^ Because the length of hospital stay has now been shortened to 2.5 days on average, this might preclude certain patients in need for prolonged acute pain treatment from optimal postoperative care. Besides continued attention for postoperative pain, nurses should be taught to recognize the occurrence of NPP from the day of surgery. Finally, another hypothesis deserving further study is whether reduction of preoperative pain by prolonged, pro-active, preoperative pain treatment can reduce acute postoperative and, subsequently, chronic pain. The rationale is the scarcity of studies on pre-emptive pain treatment starting earlier than 24 h preoperative.

Further gains in postoperative outcomes may be derived from targeting psychological risk factors pre- or immediately postoperatively. The present study again pointed to the important role of preoperative anxiety and catastrophizing, here combined in a single surgical worries variable. Brief preoperative psychological interventions, directed at diminishing anxiety and negative cognitions relating to surgery and its outcomes, may prove effective in reducing both acute postoperative pain as well as CPSP and related long-term outcomes.^[71–73]^ In addition, prehabilitation programs based on nurse counseling or physical therapy, for example, have proven successful in improving physical or mental status in different populations.^[74–76]^ For hysterectomy patients, interventions to improve their physical condition and expectations upon recovery should be assessed and selection criteria for patients who would benefit most should be established. Postoperative care after discharge can be optimized by web-based information provision to discharged women.^[77]^ Web-based survey requests for hysterectomy patients to enter their health status on a daily basis during the 1st week postoperatively might also improve postoperative (tele) monitoring despite early discharge. Concerning postoperative complications extra attention is needed for prevention of infection and urinary tract complaints.^[67,78]^ Because of the large impact of infection on recovery, preoperative counseling should pay more attention to this aspect. Finally, consensus meetings should be initiated focusing on the definition of postoperative recovery, including which outcome measure(s) to use.

## 5 Conclusion

Predictors of CPSP are moderate to high baseline levels of pain, acute postsurgical pain on day 4, and surgery-related infection. Of the preoperative psychological factors, surgery-related worries were predictive of CPSP. Overall, recovery after hysterectomy was good in terms of postoperative pain, physical functioning, and self-perceived recovery. However, 1 out of 10 patients suffered from CPSP, frequently characterized by an NPP component. Further improvements in perioperative care should aim at optimizing baseline condition and further reducing acute postsurgical pain and surgery-related infection.

## Supplementary Material

Supplemental Digital Content
