# Recovery 3 and 12 months after hysterectomy: epidemiology and predictors of chronic pain, physical functioning, and global surgical recovery: Erratum

**DOI:** 10.1097/MD.0000000000006957

**Published:** 2017-05-19

In the article, “Recovery 3 and 12 months after hysterectomy: epidemiology and predictors of chronic pain, physical functioning, and global surgical recovery”,^[1]^ which appeared in Volume 95, Issue 26 of *Medicine*, “Hospital, CzE estimate 4.667, P = 0.26, MMC 9.530, P = 0.03, OMC 7.191, P = 0.26, reference Maastricht UMC+. Type of anesthesia, GA with epidural 5.277, P = 0.56, spinal 0.407, P = 0.94, GA with spinal 58.048, P = 0.001, reference GA. Type of incision, median lower abdominal 9.200, P = 0.25, Pfannenstiel 13.493, P = 0.02, vaginal 9.582, P = 0.03, LAVH 0.760, P = 0.88, reference LH” appeared incorrectly. It should have appeared as, “Hospital, CzE estimate 4.667, P = 0.26, MMC 9.530, P = 0.03, OMC 7.191, P = 0.26, reference Maastricht UMC+. Type of anesthesia, GA with epidural 5.277, P = 0.56, spinal −0.407, P = 0.94, GA with spinal −58.048, P = 0.001, reference GA. Type of incision, median lower abdominal −9.200, P = 0.25, Pfannenstiel −13.493, P = 0.02, vaginal −9.582, P = 0.03, LAVH −0.760, P = 0.88, reference LH.”
