# Supplementary material for: Recovery 3 and 12 months after hysterectomy: epidemiology and predictors of chronic pain, physical functioning, and global surgical recovery
Source: Medicine (Baltimore). 2016 Jul 1;95(26):e3980. doi: 10.1097/MD.0000000000003980 (PMC4937912; doi:10.1097/MD.0000000000003980)
Supplement: Supplemental Digital Content [file medi-95-e3980-s001.docx]

Table 1B Baseline characteristics per subgroup CPSP and No CPSP at 3 and 12 months

|  | **3 month follow-up** | | **12 month follow-up** | |
| --- | --- | --- | --- | --- |
| **Measures** | **CPSP group** | **No-CPSP group** | **CPSP group** | **No-CPSP group** |
|  | **n = 42** | **n = 368** | **n = 34** | **n = 341** |
| Center |  |  |  |  |
| Maastricht UMC+ | 17 | 98 | 16 | 93 |
| CzE | 11 | 139 | 8 | 122 |
| MMC | 9 | 100 | 8 | 96 |
| OMC | 5 | 31 | 2 | 30 |
| Age | 45.4 (6.5) | 47.2 (7.1) | 45.8 (8.8) | 47.4 (7.1) |
| Native country NL | 36 (86%) | 350 (95%) [1] | 28 (82%) | 325 (96%) [1] |
| Marital status: living together | 38 (91%) | 315 (86%) [1] | 30 (91%) [1] | 292 (86%) [1] |
| Employment: paid job | 22 (52%) | 277 (76%) [2] | 21 (62%) | 254 (75%) [1] |
| Education: |  |  |  |  |
| no / lower | 10 (24%) | 53 (14%) | 4 (12%) | 49 (15%) |
| intermediate | 22 (52%) | 215 (59%) | 18 (53%) | 202 (59%) |
| university / higher | 10 (24%) | 99 (27%) | 12 (35%) | 89 (26%) |
| missing data | 0 | 1 | 0 | 1 |
| General health: |  |  |  |  |
| very poor / poor | 2 (5%) | 2 (1%) | 1 (3%) | 4 (1%) |
| moderate | 15 (36%) | 57 (15%) | 8 (24%) | 56 (16%) |
| very good / good | 25 (59%) | 308 (84%) | 25 (73%) | 281 (83%) |
| missing data | 0 | 1 | 0 | 0 |
| Smoking: |  |  |  |  |
| yes | 13 (31%) | 75 (21%) | 9 (27%) | 71 (21%) |
| no | 14 (33%) | 150 (41%) | 10 (29%) | 140 (42%) |
| stopped | 15 (36%) | 138 (38%) | 15 (44%) | 126 (37%) |
| missing data | 0 | 5 | 0 | 4 |
| ASA I / II / III |  |  |  |  |
| I | 23 (56%) | 200 (57%) | 22 (65%) | 190 (58%) |
| II | 16 (39%) | 148 (42%) | 10 (29%) | 132 (41%) |
| III | 2 (5%) | 4 (1%) | 2 (6%) | 4 (1%) |
| missing data | 1 | 16 | 0 | 15 |
| Body Mass Index | 26.8 (4.7) | 26.4 (4.9) | 25.7 (4.0) | 26.6 (5.0) |
| Medical history: |  |  |  |  |
| chronic disease | 18 (43%) | 131 (36%) | 15 (44%) | 120 (35%) |
| psychological problems | 14 (33%) | 103 (28%) [3] | 11 (32%) | 91 (27%) [2] |
| surgery last year | 15 (36%) | 96 (26%) [5] | 10 (29%) | 88 (26%) [2] |
| hysterectomy-related surgery last year | 10 (24%) | 64 (18%) [4] | 9 (27%) | 58 (17%) [1] |
| Gynecological history: |  |  |  |  |
| pre-menopausal state | 33 (79%) | 273 (76%) [8] | 22 (69%) [2] | 256 (76%) [4] |
| hormonal replacement therapy | 0 (0%) | 15 (4%) [3] | 1 (3%) | 13 (4%) [1] |
| contraception use | 7 (17%) | 77 (21%) [3] | 9 (27%) | 65 (19%) [2] |
| gravidity | 36 (88%) [1] | 301 (83%) [6] | 27 (82%) [1] | 283 (84%) [5] |
| parity | 1.9 (1.3) | 1.8 (1.1) | 1.6 (1.0) | 1.8 (1.1) |
| vaginal delivery or caesarean section | 35 (83%) | 300 (83%) [5] | 26 (79%) [1] | 283 (84%) [3] |
| caesarean section | 8 (19%) | 51 (14%) [2] | 4 (12%) | 46 (14%) [1] |
| sexually active | 33 (79%) | 297 (84%) [16] | 27 (79%) | 275 (84%) [15] |
| Indication (more than 1 possible): |  |  |  |  |
| leiomyoma | 12 | 120 | 15 | 106 |
| prolapse | 8 | 65 | 3 | 65 |
| menorrhagia / metrorrhagia | 22 | 172 | 18 | 163 |
| dysmenorrhoea | 4 | 22 | 2 | 18 |
| endometriosis / adenomyosis | 1 | 21 | 1 | 18 |
| abdominal pain | 3 | 23 | 6 | 17 |
| cervical dysplasia | 3 | 18 | 2 | 19 |
| other | 4 | 21 | 3 | 19 |

Table 2B Psychosocial baseline measures per subgroup CPSP and No CPSP at 3 and 12 months.

|  | **Sample 3 month follow-up** | | **Sample 12 month follow-up** | |
| --- | --- | --- | --- | --- |
| **Baseline measures** | **CPSP group** | **No-CPSP group** | **CPSP group** | **No-CPSP group** |
|  | **N = 42** | **N = 368** | **N = 34** | **N = 342** |
| Expected pain after 4 days (0-10) | 6 (4-7) | 5 (3-6) | 6 (3-7) | 5 (3-6) |
| Expected pain after 4 days NRS ≥4 | 33 (85%) [3] | 260 (72%) [5] | 25 (74%) | 243 (73%) [8] |
| Expected GSR % after 3months | 89.9 (11.5) | 93.8 (10.3) | 90.9 (15.5) | 93.1 (11.0) |
| Expected number of days until full activities | 47 (40) | 44 (30) | 47 (26) | 43 (32) |
| Expectation hysterectomy: |  |  |  |  |
| relief | 36 (88%) | 268 (74%) | 28 (82%) | 248 (74%) |
| neutral | 3 (7%) | 87 (24%) | 3 (9%) | 81 (24%) |
| loss | 2 (5%) | 7 (2%) | 3 (9%) | 7 (2%) |
| missing data | 1 | 6 | 0 | 5 |
| Expectation less femininity: |  |  |  |  |
| not at all / a bit less | 38 (95%) | 358 (98%) | 33 (97%) | 331 (98%) |
| rather much less / very much less | 2 (5%) | 7 (2%) | 1 (3%) | 7 (2%) |
| missing data | 2 | 3 | 0 | 3 |
| Surgical fear short term (0-40) | 17.8 (10.8) | 15.6 (9.7) | 21.3 (10.6) | 15.5 (9.8) |
| Surgical fear long term (0-40) | 12.1 (8.7) | 9.0 (8.4) | 12.8 (10.6) | 9.2 (8.3) |
| PCS (0-52) | 15.2 (8.6) | 11.7 (8.1) | 17.7 (9.0) | 11.5 (8.1) |
| LOT-R (0-24) | 17.7 (3.8) | 17.0 (3.7) | 16.8 (3.9) | 17.0 (3.6) |
| MOS-SSS number of persons | 12.0 (10.9) | 9.7 (6.2) | 9.4 (6.9) | 10.2 (7.2) |
| MOS-SSS total (0-100) | 89.4 (14.0) | 85.5 (18.5) | 87.7 (15.8) | 85.6 (18.6) |
| CES-D (0-60) | 14.1 (9.0) | 10.3 (7.9) | 16.5 (9.8) | 10.2 (7.8) |
| WBQ energy (0-12) | 6.1 (2.7) | 7.4 (2.7) | 6.2 (3.0) | 7.4 (2.7) |
| WBQ positive well-being (0-12) | 7.0 (2.7) | 7.8 (2.9) | 6.9 (2.7) | 7.7 (2.9) |
| History of physical abuse | 4 (10%) | 44 (12%) [3] | 5 (15%) | 35 (10%) [3] |
| History of sexual abuse | 11 (27%) [1] | 64 (18%) [6] | 8 (25%) [2] | 66 (20%) [3] |

CPSP: chronic postsurgical pain, at 3 and 12 months, hysterectomy-related highest pain last week, cut-off NRS ≥ 4. At 3 month n = 412 but because of 2 cases with missing data on the variable defining CPSP, n of the subgroups with and without CPSP count up to 410. At 12 months n = 376 but because of 1 case with missing data on the variable defining CPSP, n of the subgroups with and without CPSP count up to 375.

Expected pain data are given as median (IQR), all others as mean (sd) or number (%), [missing data].

GSR %: Global surgical recovery index, 0-100%; PCS: pain catastrophizing scale; LOT-R: life orientation test-revised; MOS-SSS: Medical Outcomes Study-social support survey with number of close friends/relatives and total score of the subscales tangible support, emotional/informative support, affective support, and positive social interaction; CES-D: Center for Epidemiological Studies - Depression; WBQ: well-being questionnaire, energy and positive well-being subscales.

Table 3B Surgery and acute pain per subgroup CPSP and No CPSP at 3 and 12 months

|  | **Sample 3 month follow-up** | | **Sample 12 month follow-up** | |
| --- | --- | --- | --- | --- |
| **Measures** | **CPSP group** | **No-CPSP group** | **CPSP group** | **No-CPSP group** |
|  | **n = 42** | **n = 368** | **n = 34** | **n = 341** |
| Anesthesia |  |  |  |  |
| GA | 36 (86%) | 306 (83%) | 29 (85%) | 283 (83%) |
| GA & epidural | 0 (0%) | 14 (4%) | 2 (6%) | 11 (3%) |
| spinal | 5 (12%) | 45 (12%) | 3 (9%) | 44 (13%) |
| GA & spinal | 1 (2%) | 3 (1%) | 0 (0%) | 3 (1%) |
| duration (hours) | 2:14 (0:44) | 2:18 (0:55) | 2:16 (0:45) | 2:15 (0:52) |
| Hysterectomy |  |  |  |  |
| total | 39 (93%) | 348 (95%) [2] | 31 (91%) | 323 (95%) [2] |
| oophorectomy | 2 (5%) | 31 (8%) | 1 (3%) | 28 (8%) |
| prolapse surgery | 8 (20%) [1] | 69 (20%) [21] | 2 (6%) [1] | 70 (22%) [17] |
| Incision |  |  |  |  |
| median lower abdominal | 3 (7%) | 19 (5%) | 1 (3%) | 18 (5%) |
| Pfannenstiel | 4 (10%) | 45 (12%) | 6 (18%) | 40 (12%) |
| vaginal | 19 (45%) | 148 (40%) | 14 (41%) | 144 (42%) |
| LH | 7 (17%) | 76 (21%) | 7 (20%) | 66 (19%) |
| LAVH | 9 (21%) | 80 (22%) | 6 (18%) | 73 (22%) |
| Blood loss (ml) | 202 (180) | 284 (381) | 262 (542) | 284 (358) |
| Training gynecologist (years) | 14.3 (10.3) | 14.1 (9.6) | 16.6 (11.3) | 13.9 (9.6) |
| Pain PACU |  |  |  |  |
| at rest (0-10) | 4.0 (1.0-7.0) | 3.0 (0.0-5.0) | 3.0 (0.0-6.0) | 3.0 (0.0-5.0) |
| at rest NRS ≥4 | 22 (56%) [3] | 131 (40%) [43] | 14 (47%) [4] | 128 (42%) [36] |
| Pain postoperative day 4 |  |  |  |  |
| highest pain last 24 hrs (0-10) | 5 (3-6.8) | 3 (1-4) | 3 (1.3-6.0) | 3 (1-5) |
| highest pain last 24 hrs NRS ≥4 | 26 (72%) [6] | 114 (35%) [45] | 13 (46%) [6] | 110 (37%) [43] |
| average pain last 24 hrs (0-10) | 4 (2-5) | 2 (1-3) | 3 (1-5) | 2 (1-3) |
| average pain last 24hrs NRS ≥4 | 24 (63%) [4] | 69 (21%) [45] | 12 (41%) [5] | 72 (24%) [42] |
| DN4 ≥3 | 19 (53%) [6] | 58 (19%) [64] | 11 (38%) [5] | 61 (22%) [59] |
| Using pain medication postoperative day 4 | 28 (76%) [5] | 226 (70%) [47] | 25 (81%) [3] | 211 (71%) [45] |

CPSP: chronic postsurgical pain, at 3 and 12 months, hysterectomy-related highest pain last week, cut-off NRS ≥ 4. At 3 month n = 412 but because of 2 cases with missing data on the variable defining CPSP, n of the subgroups with and without CPSP count up to 410. At 12 months n = 376 but because of 1 case with missing data on the variable defining CPSP, n of the subgroups with and without CPSP count up to 375.

Numbers are mean (sd), median (IQR), or number (%), [missing data].

GA: general anesthesia. Hysterectomy total: compared with subtotal hysterectomy. LH: laparoscopic hysterectomy, LAVH: laparoscopic assisted vaginal hysterectomy.

Training gynecologist: years of experience including residentship of the 1st responsible gynecologist.

PACU: Post Anesthesia Care Unit. Pain 1 hour after arrival at PACU.

DN4: neuropathic pain questionnaire, 7 self-report items (DN4-interview).

Table 4B Preoperative pain and physical characteristics per subgroup CPSP and No CPSP at 3 and 12 months

|  | **Sample 3 month follow-up** | | **Sample 12 month follow-up** | |
| --- | --- | --- | --- | --- |
| **Baseline measures** | **CPSP group** | **No-CPSP group** | **CPSP group** | **No-CPSP group** |
|  | **n = 42** | **n = 368** | **n = 34** | **n = 341** |
| Hysterectomy-related pain intensity: |  |  |  |  |
| highest pain last week (0-10) | 6.5 (4.8-8.0) | 3.0 (0.0-6.0) | 7.0 (3.0-8.0) | 3.0 (0.0-6.0) |
| highest pain last week NRS ≥4 | 33 (79%) | 169 (46%) | 25 (74%) | 158 (46%) |
| average pain last week (0-10) | 5.0 (2.8-6.3) | 2.0 (0.0-5.0) | 5.0 (2.0-6.0) | 2.0 (0.0-5.0) |
| average pain last week NRS ≥4 | 31 (74%) | 136 (37%) | 24 (71%) | 125 (37%) |
| Hysterectomy-related neuropathic pain: |  |  |  |  |
| DN4 ≥3 | 6 (16%) [4] | 32 (9%) [23] | 6 (19%) [3] | 30 (9%) [23] |
| Hysterectomy-related pain location: |  |  |  |  |
| lower abdomen | 34 (81%) | 200 (54%) | 26 (77%) | 187 (55%) |
| upper abdomen | 5 (12%) | 15 (4%) | 5 (15%) | 15 (4%) |
| back | 26 (62%) | 109 (27%) | 19 (56%) | 101 (30%) |
| legs | 10 (24%) | 38 (10%) | 9 (27%) | 35 (10%) |
| breast | 4 (10%) | 11 (3%) | 7 (21%) | 9 (3%) |
| other location | 5 (12%) | 17 (5%) | 5 (15%) | 17 (5%) |
| Onset hysterectomy-related pain since: |  |  |  |  |
| 1 week - 3 months | 4 (12%) | 32 (15%) | 1 (4%) | 31 (16%) |
| 3 - 12 months | 6 (17%) | 60 (28%) | 4 (15%) | 56 (28%) |
| >12 months | 25 (71%) | 124 (57%) | 22 (81%) | 112 (56%) |
| NA | 7 | 152 | 7 | 142 |
| Intermittence hysterectomy-related pain: |  |  |  |  |
| brief/short | 4 (12%) | 15 (7%) | 0 (0%) | 16 (8%) |
| periodic/recurrent | 20 (57%) | 147 (69%) | 15 (58%) | 137 (70%) |
| constant | 11 (31%) | 51 (24%) | 11 (42%) | 44 (22%) |
| NA | 7 | 155 | 8 | 144 |
| Non-hysterectomy-related pain intensity: |  |  |  |  |
| highest pain last week (0-10) | 5.5 (0.0-7.0) | 0.0 (0.0-5.0) | 3.5 (0.0-6.3) | 0.0 (0.0-5.0) |
| highest pain last week NRS ≥4 | 24 (57%) | 111 (30%) | 17 (50%) | 110 (32%) |
| average pain last week (0-10) | 3.5 (0.0-5.3) | 0.0 (0.0-3.0) | 2.0 (0.0-6.0) | 0.0 (0.0-3.0) |
| average pain last week NRS ≥4 | 21 (50%) | 79 (22%) | 14 (41%) | 78 (23%) |
| Non-hysterectomy-related neuropathic pain: |  |  |  |  |
| DN4 ≥3 | 7 (18%) [4] | 28 (8%) [18] | 7 (24%) [5] | 22 (7%) [17] |
| Non-hysterectomy-related pain location: |  |  |  |  |
| lower abdomen | 8 (19%) | 21 (6%) | 7 (21%) | 19 (6%) |
| upper abdomen | 1 (2%) | 11 (3%) | 2 (6%) | 9 (3%) |
| back | 14 (33%) | 50 (14%) | 7 (21%) | 53 (16%) |
| legs | 9 (21%) | 39 (11%) | 10 (29%) | 37 (11%) |
| breast | 5 (12%) | 9 (2%) | 4 (12%) | 8 (2%) |
| arms | 7 (17%) | 28 (8%) | 6 (18%) | 28 (8%) |
| shoulders | 10 (24%) | 64 (17%) | 10 (29%) | 57 (17%) |
| head | 7 (17%) | 27 (7%) | 5 (15%) | 26 (8%) |
| other location | 4 (10%) | 15 (4%) | 1 (3%) | 17 (5%) |
| BPI pain interference (0-10) | 4.4 (2.8-5.7) | 2.0 (0.1-4.3) | 4.3 (2.1-6.2) | 2.0 (0.1-4.4) |
| BPI pain interference NRS ≥4 | 23 (56%) [1] | 105 (30%) [17] | 19 (56%) | 102 (31%) [16] |
| Using pain medication | 25 (63%) [2] | 149 (42%) [13] | 21 (62%) | 139 (43%) [14] |
| Other pain treatment | 1 (3%) [2] | 32 (9%) [8] | 3 (9%) [1] | 24 (7%) [9] |
| Other physical baseline measures |  |  |  |  |
| SF36 physical activity (0-100) | 72.4 (23.9) | 83.7 (18.2) | 74.9 (24.5) | 83.0 (18.9) |
| Active days / week, (0-7) | 4.5 (2.4) | 4.3 (2.2) | 3.9 (2.2) | 4.3 (2.2) |

CPSP: chronic postsurgical pain, at 3 and 12 months, hysterectomy-related highest pain last week, cut-off NRS ≥ 4. At 3 month n = 412 but because of 2 cases with missing data on the variable defining CPSP, n of the subgroups with and without CPSP count up to 410. At 12 months n = 376 but because of 1 case with missing data on the variable defining CPSP, n of the subgroups with and without CPSP count up to 375.

Numbers are median (IQR), number (%), [missing data]. NA: not applicable.

DN4: neuropathic pain questionnaire, 7 self-report items (DN4-interview).

Table 5B Final multivariate models: additional legend concerning data reduction and analysis

To maintain a certain uniformity across multivariate analyses of the three outcome measures and to reduce the number of variables, for predictors with a high level of similarity or overlap an a priori choice was made regarding which one to retain. For preoperative health status we chose to use ASA classification instead of the general health status, body mass index, or the presence of chronic disease as obtained from the comorbidity screening list, because the ASA classification is readily available and clinically useful. For preoperative pain status both highest hysterectomy-related and highest non-hysterectomy-related pain (NRS) were used rather than preoperative neuropathic pain, pain interference, or pain treatment. The reason was that these variables do not overlap and best match the primary outcome variable which was also defined as the highest (hysterectomy-related) pain at 3 months. Acute postsurgical pain on day four was entered using neuropathic pain and not by NRS score or pain treatment because NPP contributed most to statistical model improvement. If bivariately significant, pain immediately after surgery, at the Post Anesthesia Care Unit (PACU), was also entered in the model. Of the postoperative complications only surgery-related infection was assessed for predictor analysis because this was assessed explicitly, in contrast to other complications, which were assessed more generally.

The following variables were significant at p = .10 level, but not entered in the multivariate model because of the a priori selection of predictor variables described above: baseline general health status, physical functioning, expected global surgical recovery, SFQ long-term, PCS, CES-D, WBQ-energy, hysterectomy-related baseline pain location, non-hysterectomy-related baseline NPP and pain location, baseline pain interference, baseline analgesia use, and acute pain NRS day 0-4 with regard to the outcome CPSP. General health status, number of active days/week, expected global surgical recovery, expected number of days until reuptake of full activities, SFQ short- and long-term, CES-D, both W-BQ12 subscales, hysterectomy-related baseline pain location, non-hysterectomy-related baseline NPP and pain location, pain interference, analgesia use postoperative day 0, 2, 3, and acute pain NRS day 2-4 were the variables significant at p .10 but not entered for outcome physical functioning. And finally, the expected number of days until reuptake of full activities, SFQ short-term, PCS, LOT-R, CES-D, W-BQ12 positive well-being subscale, expected pain, hysterectomy-related and non- related baseline pain location, pain interference, analgesia use at postoperative day 1-4 and acute pain NRS day 1-4 were not entered for the outcome global surgical recovery.

To further reduce the load of bivariate significant predictors on the multivariate model an intermediate step was made. Based on domain or point in time, predictors were clustered in six blocks. Block 1 consisted of the control variables hospital, age, type of incision, type of anesthesia, baseline measure or expectation of the outcome, and a time variable defining 3 and 12 month follow-up. Block 2 consisted of predictors concerning baseline health status and sociodemographic variables, block 3 consisted of gynecological history and indication for surgery, block 4 concerned psychosocial predictors, block 5 surgery and acute pain, and block 6 represented self-reported infection during follow-up. Bivariate significant predictors of each of the blocks 2-6 were added separately to the control variables of block 1, and backward analyses were applied to select which variables of each block were selected (criterion p < .10) for the final multivariate analyses. Two multivariate models are presented. Model 1, consisting of the predictors of block 1-4, reflects the impact of control variables and preoperative predictors. In model 2 the per- and postoperative variables of block 5 and 6 were added.

For outcome pain, bivariate analyses were performed using a random intercept model with a variance components covariance structure. If the variance of the random intercept was close to zero, analyses were repeated without random intercept. Eventually this applied to all potential predictors except sexual activity, number of close friends/relatives, and acute pain at the PACU. As a result, the multivariate analyses were also performed without random intercept.

Table 6 Health care visits for self-reported complications during 3 and 12 month follow-up

|  | **Sample 3 month follow-up** | | **Sample 12 month follow-up** | |
| --- | --- | --- | --- | --- |
| **Visits** | **CPSP group**  **n = 42** | **No-CPSP group**  **n = 368** | **CPSP group**  **n = 34** | **No-CPSP group**  **n = 341** |
| Visit general practitioner | 15 (36%) | 70 (19%) | 12 (35%) | 47 (14%) |
| Visit specialist | 20 (48%) | 66 (18%) | 13 (38%) | 39 (11%) |
| Visit emergency | 4 (10%) | 17 (5%) | 3 (9%) | 9 (3%) |
| Reason visit: |  |  |  |  |
| infection / fever | 16 (38%) | 43 (12%) | 5 (15%) | 15 (4%) |
| urinary tract | 8 (19%) | 39 (11%) | 1 (3%) | 15 (4%) |
| abdominal / gastrointestinal complaints | 9 (21%) | 31 (8%) | 8 (24%) | 16 (5%) |
| pain / cramp | 11 (26%) | 22 (6%) | 6 (18%) | 20 (6%) |
| cystitis | 5 (12%) | 25 (7%) | 0 (0%) | 7 (2%) |
| bleeding / haematoma | 9 (21%) | 19 (5%) | 3 (9%) | 7 (2%) |
| musculoskeletal complaints | 7 (17%) | 13 (4%) | 6 (18%) | 17 (5%) |
| wound inspection | 2 (5%) | 15 (4%) | 5 (15%) | 1 (<1%) |
| ileus / obstipation | 2 (5%) | 12 (3%) | 1 (3%) | 2 (<1%) |
| Hospitalized (no surgery) | 2 (5%) | 8 (2%) | 0 (0%) | 3 (1%) |

CPSP: chronic postsurgical pain, at 3 and 12 months, hysterectomy-related highest pain last week, cut-off NRS ≥ 4. At 3 month n = 412 but because of 2 cases with missing data on CPSP outcome, n of the subgroups with and without CPSP count up to 410. At 12 months n = 376 but because of 1 case with missing data on CPSP outcome, n of the subgroups with and without CPSP count up to 375.

Number (%).
